# Determining the roots of Urnfield Culture at Přáslavice, Czech Republic

**DOI:** 10.1007/s12520-026-02436-2

**Published:** 2026-04-07

**Authors:** Christina Cheung, Klára Šabatová, Ivana Jarošová, Kévin Salesse, Hannah F. James, Jacob I. Griffith, Zdeněk Tvrdý, Christophe Snoeck

**Affiliations:** 1 Department of Anthropology, Chinese University of Hong Kong, Shatin, Hong Kong; 2 Centre for Archaeology and Cultural Heritage Studies, Chinese University of Hong Kong, Shatin, Hong Kong; 3 Archaeology, Environmental Changes & Geo-Chemistry Research Group, Vrije Universiteit Brussel, Pleinlaan 2, Brussels, 1050 Belgium; 4 Department of Archaeology and Museology, Faculty of Arts, Masaryk University, Arna Nováka 1, Brno, 60200 Czech Republic; 5 Department of Anthropology, Faculty of Science, Masaryk University, Kotlářská 2, Brno, 611 37 Czech Republic; 6 Anthropos Institute, Historical Museum, Zelný trh 6, Moravian Museum, Brno, 65937 Czech Republic

**Keywords:** Urnfield Culture, Moravia, Mobility, Strontium isotopes

## Abstract

This study examines the residential mobility patterns and funerary practices of an Urnfield cemetery at Přáslavice, Czech Republic, using radiocarbon dating, carbon, oxygen, and strontium isotope analyses, and FTIR analysis. A regional bioavailable strontium baseline map was created using strontium isotope ratios (^87^Sr/^86^Sr) of modern plant samples (n=208) from 70 localities across Moravia and Czech Silesia. Radiocarbon dates (n=17) suggest that the cemetery was primarily in use during the Late Bronze Age (1300–1100 BCE), with some burials dated to the Final Bronze Age (1100–800 BCE). Most of the Late Bronze Age burials have homogenous ^87^Sr/^86^Sr values (0.7114–0.7128) well within the established local Sr baseline, thus supporting the hypothesis that Urnfield at Přáslavice was a local development. FTIR and carbon and oxygen isotope analyses also suggest the group practiced cremation similarly to other Urnfield populations. Overall, this study not only enhances our knowledge of the social dynamics and funerary practices at Přáslavice, but also establishes a crucial bioavailable Sr baseline for future palaeomobility studies in the region.

**Supplementary Information:**

The online version contains supplementary material available at 10.1007/s12520-026-02436-2.

## Introduction

**Fig. 1:**
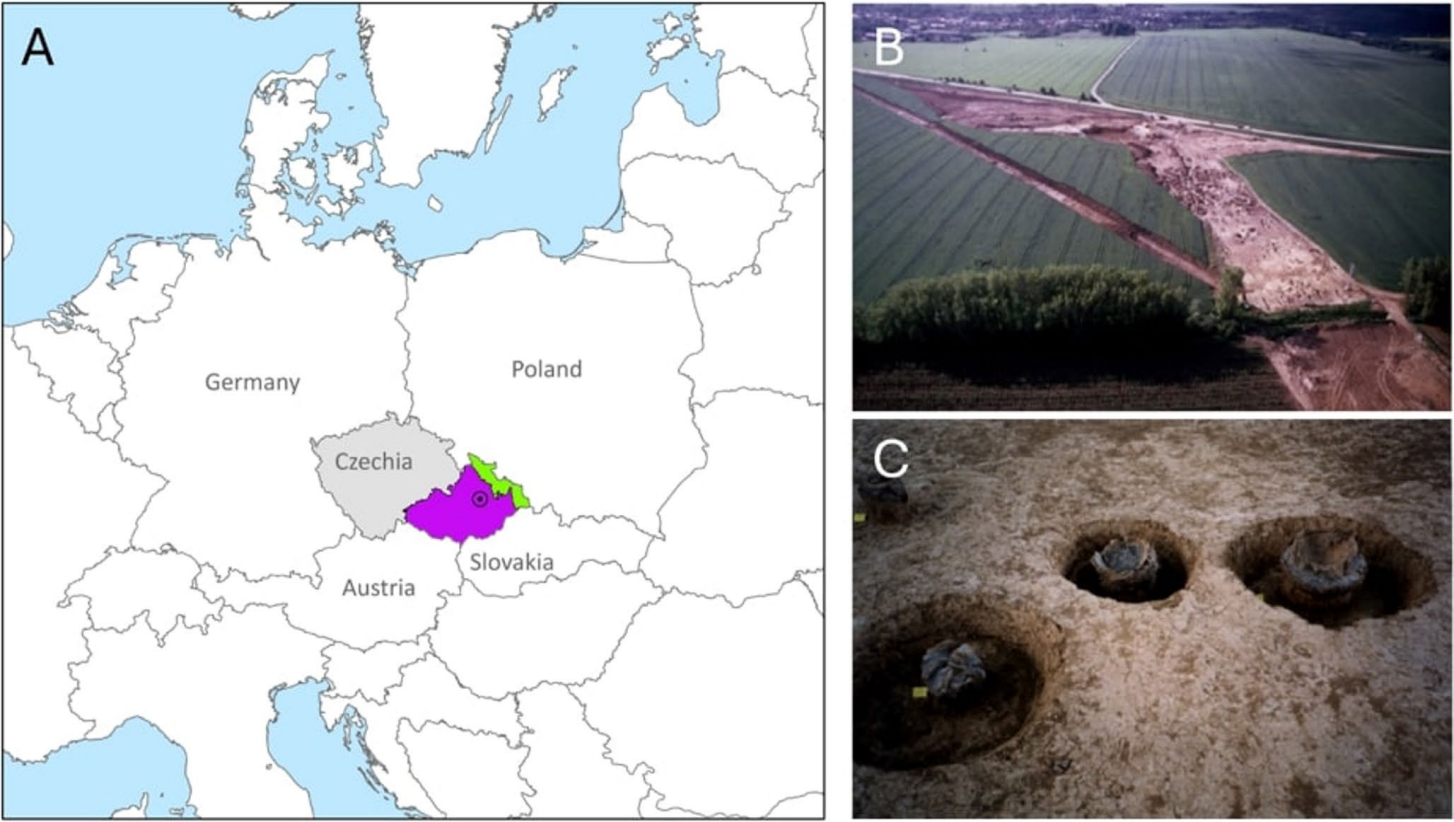
A. Location of Přáslavice within Czechia, with the historical regions of Moravia and Czech Silesia indicated by purple and green respectively; B: bird’s-eye view of the site; C: a close-up image of urned burials from the site. Photos of the site are reproduced with permission from the Archaeological Center Olomouc

The Urnfield Culture is a Late (1300–1100 BCE) and Final (1100–800 BCE) Bronze Age culture widespread in Central Europe. Between c. 1300 and 800 BCE, this culture was prominent in the Danube-Carpathian Basin region, but was also found in the Elbe, Odra, and Wisla basins (Buchvaldek et al. 2007; Harding and Fokkens 2013; Cavazzuti et al. 2019). The Urnfield Culture is identified by its distinctive funerary practice, where cremated remains of their dead were placed in cremation graves, mostly in urns; while most burials from the preceding culture, the Tumulus Culture (Middle Bronze Age, c. 1600–c. 1300 BCE), were inhumations or cremations placed under tumuli (Harding 2000; Falkenstein 2012; Jiráň et al. 2013b). A key question asked by archaeologists concerns that of the nature of the transition between the two cultures: did the Urnfield Culture represent a migratory population, or was it a local development? To answer this question, archaeologists have been looking for a site where evidence of a cultural transition is present. The Late and Final Bronze Age burial site located at Přáslavice, in the Olomouc district of the modern Czech Republic (Fig. 1), presents such exact opportunity. Although only cremated burials from the Urnfield Culture were found, the cemetery is associated with a small village that has evidence of both Tumulus and Urnfield material cultures. Additionally, some of the burials contain artefacts belonging to the transitional phase between Tumulus and Urnfield Cultures, thus providing a unique opportunity to investigate the transition of the two cultures in this area.

This study focuses on the Urnfield burials from Přáslavice, by examining the residential mobility of the cremated remains (n=32) found at the site using strontium isotope analysis (^87^Sr/^86^Sr). In parallel, the way in which these individuals were cremated is investigated through Fourier Transform Infrared (FTIR) Spectroscopy and carbon and oxygen isotope analyses (see Salesse et al. 2021 and Stamataki et al. 2021 for more details). Radiocarbon dates have been obtained from 17 of the burials to better identify temporal trends, if any, amongst the population. In addition, 208 modern plant samples from 70 localities across Moravia and Czech Silesia are also analysed to help establish the local and regional bioavailable Sr (BASr) baselines. There are two main objectives to this study: i) to reconstruct the mobility patterns and funerary practices, and by extension, social dynamics of the Urnfield population at Přáslavice; and ii) to provide a detailed BASr isoscape of the region. 

## Background

### Archaeological background

The transition from Tumulus to Urnfield Culture has sometimes been referred to as an “epoch-making cultural change” (Falkenstein 2012). This transition has brought more than just new funerary practices, but also signified complex and significant social and geo-political changes across Europe during this time (Cavazzuti et al. 2022). While the nature of this transition has been studied widely, in Moravia, most researchers believed that Urnfield Culture was a local development (Říhovský 1982; Nekvasil 1987; Štrof 1993; Šabatová 2020). Although, a few argued that it was an external culture, possibly arriving from the north (Bouzek 2010; Jiráň et al. 2013a; Juchelka 2014; Horňák 2016). At Přáslavice, a clear transition in material culture from Tumulus to Urnfield has been observed at the residential area, thus supporting the hypothesis that the Urnfield Culture was a local development here (Šabatová 2006, 2020). This study aims to use geochemical methods to further investigate the matter. Since no burial has been found for the Tumulus period, this study focuses on the Urnfield phase at the site.

The Urnfield Culture (c. 1300–800 BCE; Late to Final Bronze Age) in Central Europe is characterised by dense settlements with strong evidence of well-established agricultural and husbandry practices (Gedl 1975; Štrof 1993; Smrž 1998; Roblíčková 2003; Hajnalová 2012; Jiráň et al. 2013a, 2013b; Vránová 2013; Pokorná et al. 2024). Intensive landscape archaeological surveys suggested that small-scale regional mobility (between 0.5 and 2 km) were common amongst smaller settlements along rivers, such as Přáslavice (Smrž 1998; Jiráň et al. 2013a; Vránová 2013). The largest Urnfield burial grounds in Central Europe, often containing thousands of burials, were primarily associated with the Lusatian Culture – a regional culture within the broader Urnfield Culture tradition. These large burial grounds indicate long-term settlement histories and use of cemeteries (Plesl and Hrala 1987; Harding and Fokkens 2013).

The burial site at Přáslavice (49°35’10.6"N, 17°22’07.1"E) contains at least 106 graves. It is one of the largest excavated Late Bronze Age burial sites from the Lusatian Urnfield Culture in Moravia after Moravičany (Nekvasil 1982). Přáslavice is also associated with the largest excavated settlement of this time in Moravia (Šabatová 2007a; Šabatová 2007b; Šabatová and Vitula (2002). Discovered as part of a development-led excavation during the 1990 s, the site is situated 8 km east of the modern city of Olomouc (Fig. 1). Funerary practices at Přáslavice follow those of the Lusatian Urnfield tradition, where funerary furniture is largely similar to those of other Urnfield Cultures, except with the notable paucity of metal grave goods (Nekvasil 1987; Šabatová 2007b; Liczbińska et al. 2020; Šumberová et al. 2021) and little social stratification (Nekvasil 1978; Veliačik 1983; Štrof 1993).

At Přáslavice, based on the stylistic analysis of the grave goods, it is generally believed that the burial ground was established during the Early Urnfield period/Late Bronze Age (c. 1300 − 1100 BCE; Šabatová and Vitula 2002). Two earlier published dates from charcoal samples (H1 [VERA 587]: 2955 ± 45 BP; H14 [VERA 588]: 2990 ± 40 BP) further confirmed the site belonged to the Late Bronze Age (1386–1056 cal. BCE) (Šabatová and Vitula 2002). To confirm the periodisation of the site, radiocarbon dates have been obtained from an additional 17 samples in this study.

The cemetery has been excavated in three areas: A (3 graves), B (57 graves), and C (46 graves) (see layout of excavation area in S1). The space between these areas has not been excavated. Spatial distribution of the burials suggested there may be several burial groups, which is a typical phenomenon in Urnfield cemeteries (Dohnal 1974; Nekvasil 1978; Štrof 1993; Šumberová et al. 2021). The centres of some of these groups are empty, and it is assumed that graves in the centres were placed on the surface, covered by mound embankments (Nekvasil 1978; Štrof 1993). Of the 106 burials at Přáslavice, 48 cremations are preserved. Samples from fully calcined long bones were taken from a total of 32 cremation burials for carbon, oxygen, strontium isotope analysis, and FTIR measurements. For four of the burials, an additional tooth was further selected (three enamel fragments and dentine from one fully calcined root) to monitor possible mobility during life (Taylor et al. 2020; Cavazzuti et al. 2021; Veselka et al. 2021; Frère et al. 2025).

### Geographical background

Přáslavice is located in a rolling landscape at the edge of the most fertile part of the area (Demek 1992; Tyráček et al. 1994), enjoying a relatively warm and humid continental climate. The region is part of the geological unit Moravian-Silesian Palaeozoic, which is a subdivision of the Bohemian Massif. It is a geomorphological feature located on the Tršice benchland on the Oder Mountains foreland. The Moravian basins, a region on the west, connect the Bohemian Massif with the Western Carpathians through the Morava River basin. The southeast area features northern part of the Carpathian foredeep, the Moravian Gate, following the river Bečva’s basin.

### Reconstructing past mobility patterns using strontium isotope analyses of cremated human remains

Strontium is an alkaline earth element, with four naturally occurring stable isotopes, one of which (^87^Sr) is radiogenic and formed through the radioactive β-decay of Rubidium-87 (^87^Rb). As such, the ^87^Sr/^86^Sr of different types of bedrock vary depending on their age and their initial Rb content (Faure and Powell 1972). This strontium is then transferred into the soil, and the bioavailable fraction is picked up by plants which are then eaten by animals and humans. This strontium (together with strontium from other sources – i.e. dust, rainfall, seaspray, etc.) can substitute for calcium in bones and teeth (LeGeros et al. 1986; Wopenka and Pasteris 2005). By measuring the strontium isotope ratios in animal and humans, it then becomes possible to obtain information of the origins of their foods and drinks (Montgomery 2010). With adequate BASr baseline, improved interpretations of palaeomobility are possible (see Holt et al. 2021).

Strontium isotope analyses are now routinely conducted on cremated human remains that are fully calcined and adequately pre-treated, offering new insights into mobility and landscape use in different periods and regions (Snoeck et al. 2015; Snoeck et al. 2022). Multiple studies focusing on the Urnfield period and employing similar techniques have been published (Cavazzuti et al. 2021; Sabaux et al. 2021; Cavazzuti et al. 2022; Capuzzo et al. 2024; Fritzl et al. 2024). In most cases, the ^87^Sr/^86^Sr results suggest a majority of those buried in Urnfields were local, with only a few outliers that could be coming from further afield. To examine past mobility patterns at Přáslavice, a bioavailable strontium (BASr) baseline is required. Plants are considered one of the best proxy for BASr baselines as they source strontium from soils and water and form the base of the terrestrial food chain (Holt et al. 2021; Spies et al. 2025). By measuring strontium isotope ratios on cremated remains from Přáslavice and creating the first BASr baseline for the area around the burial site, new insights on the supra-regional relationships between Urnfield groups can be addressed.

### Reconstructing cremation practices using C and O isotope analyses and FTIR

Previous experiments have shown that wood-derived carbon plays a dominant role in the chemical exchanges that occur in the cremation environment, and significantly influences the δ^13^C signature of CO_2_ present in the cremation atmosphere. In addition, these studies highlighted that most but not all the carbon in the carbonate fraction of calcined bones is replaced by carbon from the fuel (Hüls et al. 2010; Zazzo et al. 2012; Snoeck et al. 2014a; Salesse et al. 2021). Moreover, biogenic δ^18^O values in bone carbonate are primarily determined by the δ^18^O values of locally consumed water, which are shaped by a set of geophysical factors (Longinelli 1984; Schwarcz et al. 1991; Bowen and Wilkinson 2002). During cremation, these biogenic δ^18^O values will shift towards, if not be entirely replaced by, those of the surrounding atmosphere, which is severely depleted in ^18^O due to the combustion of wood (Snoeck et al. 2014b; Snoeck et al. 2016b; Salesse et al. 2021). The δ^18^O values of wood result from a combination of factors: the δ^18^O signature of soil water, evaporative processes in transpiring leaves, and isotopic exchange between oxygen atoms in organic molecules and the local water within the cells where these molecules are produced (Babour 2007).

At the same time, depending on the pyre temperature and the duration of the cremation, the crystalline structure of bioapatite changes. These changes can be monitored through infrared spectroscopy (Lebon et al. 2010; Thompson et al. 2013; Snoeck et al. 2014b). Thus, the combination of carbon and oxygen isotope analyses with FTIR offers the opportunity to gain insights into the way individuals were cremated (Salesse et al. 2021) and see if the practices were similar or more variable between individuals, sites, and regions. At this point, only limited data is available for the Urnfield period (Belgium and Northern Italy) (Stamataki et al. 2021; Capuzzo et al. 2024; Frère et al. 2025). By expanding this line of research to Přáslavice, new regional trends can be gained in the way cremation was performed during the Late Bronze Age.

## Materials and methods

### Materials

#### Materials – archaeological human samples

Cremated remains were sampled from a total of 32 burials from the Přáslavice cemetery. Samples were selected primarily from individuals where some osteological (e.g. age and/or sex categories) and archaeological information (e.g. burial practices) is available. Of the analysed samples, one comes from area A, 16 come from area B, and 15 come from area C. Based on osteological analysis of the cremated burials, 26 of the samples come from single burials and seven come from multiple burials. Regardless of the number of individuals osteologically identified, only one long bone fragment was selected from each burial, which was then used for all analyses (including radiocarbon dating for 17 of these long bones – see Sabaux et al. 2021; Sabaux et al. 2024). Four of the burials (H35, H36, H37, and H46) have teeth associated with them. Therefore, an additional tooth was also sampled from each of these individuals. For H35, H36, and H46, enamel from a molar or premolar was used. For H37, only a fully calcined tooth root (unidentified) was available for sampling, as the enamel was not preserved. The root was large enough to be divided into two sections: CC077-R1 refers to the part closer to the cervix, where CC077-R2 refers to the part including the apex. As none of these teeth can be fully identified, it is not possible to pinpoint the formation time of the dental tissues. However, the formation periods of these tissues should still precede those of the bones, therefore reflecting mineral uptake during the early lives of the individuals. All osteological and archaeological information of samples analysed is provided in S2.

#### Materials – modern plant samples

**Fig. 2:**
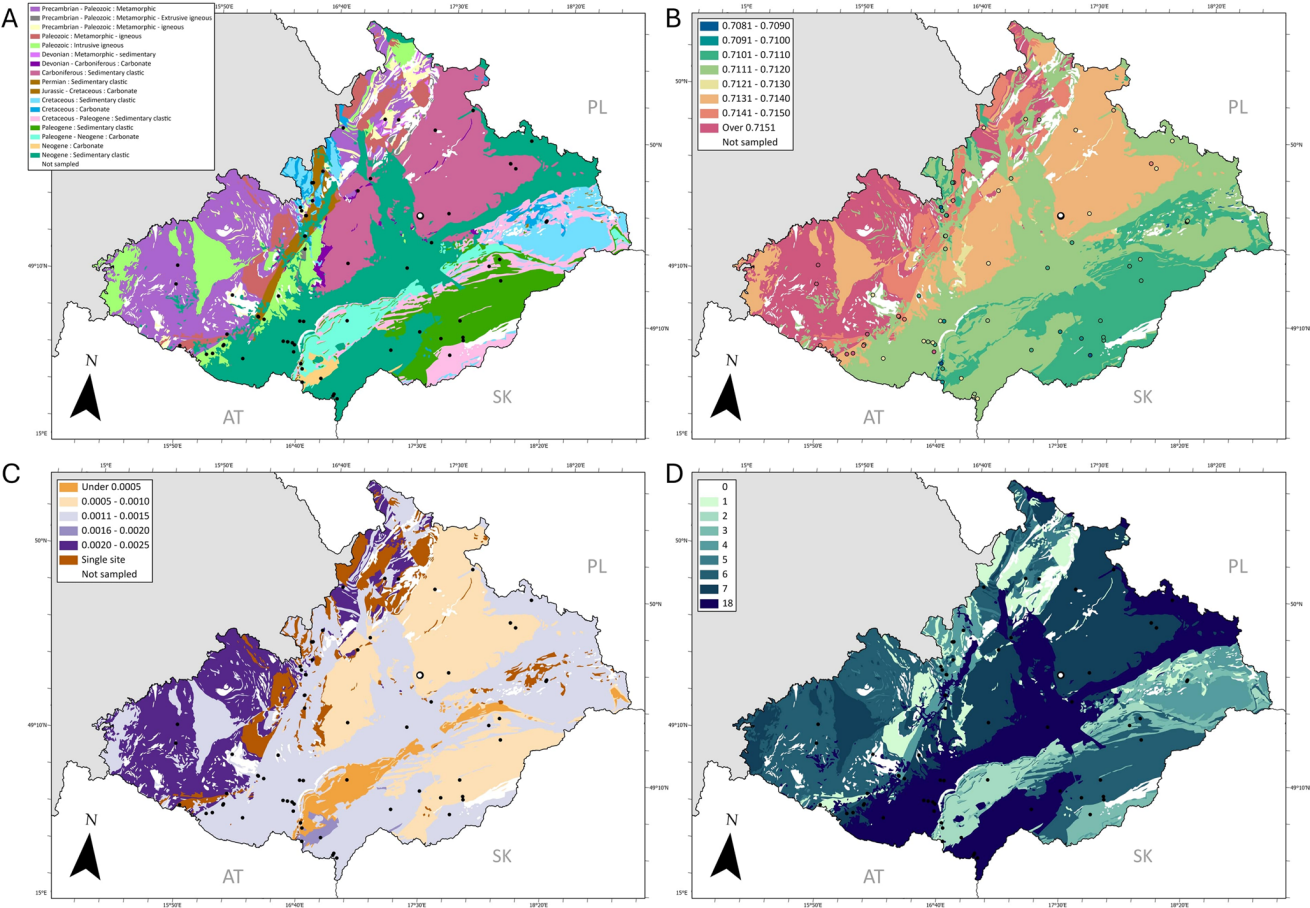
(**A**) Simplified geological map of Moravia and Czech Silesia; (**B**) Median 87Sr/86Sr per simplified geological unit; (**C**) Median Absolute Deviation (MAD) for each simplified geological unit; (**D**) number of sampling sites from each respective simplified geological unit. The location of Přáslavice is denoted by an open circle. Latitude and longitude graticules are marked on the edges of the map

To establish a BASr baseline map for Moravia and Czech Silesia, 208 modern plant remains were collected from a total of 70 localities across the region (coordinates of all the localities are provided in S3 and shown in Fig. 2). From each of the localities, plant clippings were collected from plants with three varying rooting depths: trees, shrubs, and grasses, whenever possible, following the protocol described in Snoeck et al.(2020). Studies have shown that plants with varying rooting depths would have differential dependency on aeolian, topsoil, and bedrock Sr (Reynolds et al. 2012; Willmes et al. 2018). Thus, by collecting and analysing plants of varying rooting depths, the local and regional BASr variability can be better characterised. Localities of plant samples were selected based on the following criteria: covering a variety of geological bedrocks; areas minimally disturbed by anthropogenic processes; proximity to archaeological sites; and public accessibility.

### Methods

#### Methods – Sr extraction and analytical methods

Sr extraction and analysis were performed at the AMGC (Archaeology, Environmental Changes & Geo-Chemistry) laboratories at the Vrije Universiteit Brussels (VUB), Belgium. Studies have shown that enamel and fully calcined bones are both reliable substrates for strontium isotope analyses once adequately pre-treated (Budd et al. 2000; Snoeck et al. 2015). In this study, only fully calcined bones (or tooth) and enamel were selected. These samples were treated and processed following the procedure described in Snoeck, et al. (2015), Snoeck and Pellegrini (2015), Stamataki et al. (2021), and Gerritzen et al. (2024). Modern plants were treated and processed following the procedure described in James et al. (2025). For the full description of the pretreatment and Sr extraction protocol, please refer to S4.

Extracted Sr was analysed with a Nu Plasma 3 (PD017 from Nu Instruments, Wrexham, UK) for both Sr isotopes (^87^Sr/^86^Sr) and concentrations ([Sr] – see Boonants et al. 2025). The Sr isotopic ratios were automatically normalised to ^86^Sr/^88^Sr = 0.1194 using an exponential law. To normalise all sample measurements, a standard bracketing method was employed using NIST SRM 987, with the recommended value of ^87^Sr/^86^Sr = 0.710248 (Weis et al. 2006). Procedural blanks were considered negligible, with a total Sr (V) of a maximum of 0.02 compared to 10–12 V for analyses (~ 0.2%). For each sample, the ^87^Sr/^86^Sr ratio is reported with a 2SE error (which is the absolute error of the individual sample analysis – internal error). Repeated measurements of the NIST SRM987, 1400, 1486, and 1515 resulted in ^87^Sr/^86^Sr = 0.710249 ± 0.000033 (2SD; *n* = 21), 0.713106 ± 0.000029 (2SD; *n* = 17), 0.709305 ± 0.000027 (2SD; *n* = 5), and 0.713946 ± 0.000039 (2SD; *n* = 43), respectively. These values are consistent with the reported value of 0.710252 ± 0.000013 (2SD; TIMS; Weis et al. 2006), 0.713120 ± 0.000033 (2SD; *n* = 6; Lazzerini et al. 2021), 0.709310 ± 0.000038 (2SD; *n* = 77) and 0.713957 ± 0.000035 (2SD; *n* = 233), respectively (Gerritzen et al. 2024). [Sr] of NIST SRM1400 and 1515 yielded an average value of 243 ppm ± 4% RSD (*n* = 4) and 23 ppm ± 4% RSD (*n* = 8), which are consistent with the certified value of 249 ppm ± 3% RSD and 25 ppm ± 4% RSD, respectively.

#### Methods – carbon and oxygen isotope analyses of cremated human remains

For the analysis of carbon and oxygen isotopes, all samples were analysed in duplicates. For each analysis, 15–17 mg of pretreated bone powder (see S4) was placed in sealed glass tubes (exetainer^®^ from Labco Limiter) and then flushed with helium to eliminate atmospheric components. Subsequently, 13–15 drops of phosphoric acid were added with a Sterican^®^ needle (0.90Ø x 25 mm, 20 G x 1 inch), triggering a reaction with the carbonates in the bone, resulting in the release of CO_2_. This released gas was then analysed with a Nu Perspective IRMS (Isotope Ratio Mass Spectrometer) from Nu Instruments, coupled with a Nu Gasprep automatic gas bench at AMGC, VUB. The isotope ratios are expressed as delta (δ) units, quantifying the deviation in isotope ratios from a specific standard value (McKinney et al. 1950; Hoefs 2009; Sharp 2017). The outcomes are presented as permil (‰) deviations from the VPDB reference standard. Three standards were used to calibrate the isotopic data: IAEA-603 (δ^13^C = 2.46‰ and δ^18^O = −2.4‰), IAEA-CO8 (δ^13^C = −5.76‰ and δ^18^O = −22.7‰), and IA-R022 (δ^13^C = −28.6‰ and δ^18^O = −22.7‰). The matrix matched international standard NIST SRM 1400 was used to check the stability of the instrument throughout the analytical sessions. Analytical precision was better than ± 0.40‰ for δ^13^C and ± 0.41‰ for δ^18^O (*n* = 6; 1SD).

#### Methods – infrared analyses of cremated human remains

Before FTIR-ATR measurements, pretreated bone powder (see S4) was sieved using a 50 μm woven stainless-steel mesh sieve following Kontopoulos et al. (2018). Each sample was analysed in triplicate (2–3 mg of bone powder for each measurement). Infrared analyses were conducted at AMGC, VUB using a Bruker Vertex 70v FTIR spectrometer under vacuum (spectral range: 4000–400 cm-1; number of scans: 32; spectral resolution: 4 cm-1; mode: absorbance). The software OPUS 7.5 was used to analyse the spectra. Before measuring the archaeological samples, a standard (either bone ash NIST SRM1400 or CCB01 (Lucideon)) was tested in triplicates to ensure the instrument was producing reliable results. After each measurement, the spatula, crystal plate, and anvil of the pressure applicator were cleaned with Isopropanol.

All the indices were calculated after baseline correction, as described by Stamataki et al. (2021). The infrared splitting factor (IRSF) provides information on the bone apatite crystallinity (Weiner and Bar-Yosef 1990). The carbonyl-to-carbonate ratio (C/C), and the carbonate-to-phosphate ratio (C/P) are used to compare the absorbance at two wavelengths associated with carbonates and phosphates. The hydroxyl-to-phosphate ratio (OH/P) indicates alterations in hydroxyl groups in heated bone apatite (Snoeck et al., 2014b), and the cyanamide-to-phosphate ratio (CN/P) is indicative of possible reducing conditions (Zazzo et al. 2013; Snoeck et al. 2014b; Salesse et al. 2021).

#### Methods – statistical analysis and graphics

Quantitative analyses of all the data are conducted using the statistical package R version 4.2.2 (R Core Team 2019) with R Studio (RStudio Team 2018). Data are visualised using the package “ggpubr” (Kassambara 2020). Distribution normality is tested using the Shapiro-Wilk’s method. For groups with normally distributed data and/or sufficient sample size (n greater than 10), the parametric tests, unpaired independent Student’s t tests and one-way ANOVA, are used to determine differences between group means for ^87^Sr/^86^Sr values. For groups where data are not normally distributed and/or have small sample size (*n* < 10), the non- parametric Mann-Whitney U and Kruskal-Wallis tests are used. A 0.05 probability (*p* < 0.05) is considered significant. Outliers are identified as values that fall outside the range of ± 2 standard deviations (SD).

Spatial analyses were undertaken in ArcGIS Pro 2.9.2 using the Spatial and Geostatistical Analyst extensions (eESRI). A baseline geological map, Geological map of the Czech Republic 1: 500,000 (GEOCR500), was obtained from the Czech Geological Survey. To allow for the creation of a bioavailable strontium baseline for Moravia and Czech Silesia, the ‘name’ category which contains age and lithology descriptions was simplified. Age descriptions were simplified to Period level (except Variscan, which was retained), and lithology descriptions were simplified to nine broad categories; carbonate, sedimentary clastic, extrusive igneous, intrusive igneous, metamorphic, metamorphic igneous, metamorphic extrusive igneous, metamorphic intrusive igneous, and metamorphic sedimentary (see S5). In this study, modern plant samples were taken from within 17 age lithology categories. These sampled categories represent 96% of the land surface of Moravia and Czech Silesia. Using the Summarize Within tool, the median ^87^Sr/^86^Sr for each age lithology category was calculated using the 208 modern plant samples from 70 sampling locations across Moravia and Czech Silesia (summary statistics in Table 1) (James et al. 2022).

**Table 1:** Summary statistics for modern plant samples used in the creation of the BASr map as well as the archaeological human remains

No.	Simplified Age Lithology categories	Number of samples	Mean	Median	Mean Absolute Deviation	1 st Quartile	3rd Quartile
1	Carboniferous: Sedimentary clastic	21	0.7131	0.7128	0.0010	0.7122	0.7137
2	Cretaceous - Paleogene: Sedimentary clastic	9	0.7103	0.7106	0.0009	0.7086	0.7109
3	Cretaceous: Carbonate	3	0.7107	0.7109	0.0002	0.7105	0.7109
4	Cretaceous: Sedimentary clastic	12	0.7117	0.7117	0.0009	0.7112	0.7124
5	Devonian - Carboniferous: Carbonate	3	0.7121	0.7123	0.0001	0.7120	0.7123
6	Devonian: Metamorphic - sedimentary	3	0.7115	0.7115	0.0002	0.7114	0.7116
7	Jurassic - Cretaceous: Carbonate	3	0.7089	0.7088	0.0002	0.7088	0.7090
8	Neogene: Carbonate	6	0.7110	0.7109	0.0024	0.7093	0.7125
9	Neogene: Sedimentary clastic	54	0.7118	0.7122	0.0014	0.7108	0.7127
10	Paleogene - Neogene: Carbonate	6	0.7112	0.7110	0.0004	0.7108	0.7114
11	Paleogene: Sedimentary clastic	16	0.7105	0.7102	0.0007	0.701	0.7112
12	Paleozoic: Intrusive igneous	21	0.7138	0.7144	0.0019	0.7124	0.7150
13	Paleozoic: Metamorphic - igneous	3	0.7141	0.7141	0.0003	0.714	0.7143
14	Permian : Sedimentary clastic	12	0.7148	0.7143	0.0013	0.7136	0.7155
15	Precambrian - Paleozoic : Metamorphic	18	0.7178	0.7177	0.0040	0.7149	0.7201
16	Precambrian - Paleozoic : Metamorphic - Extrusive igneous	3	0.7116	0.7117	0.0001	0.7116	0.7117
17	Precambrian - Paleozoic : Metamorphic - igneous	15	0.7114	0.7110	0.0023	0.7105	0.7130
	Human	32	0.7121	0.7122	0.0003	0.7121	0.7124

#### Methods – radiocarbon dating

All samples were sent to the Royal Institute for Cultural Heritage (RICH, Brussels, Belgium) for radiocarbon dating. At RICH, cremated samples undertook pre-treatment and graphitisation following the standard procedure outlined in van Strydonck et al. (2009), samples were then measured on a Micadas (Mini Carbon Dating System) AMS (Boudin et al. 2015).

To ensure data reliability, for two samples (H31 and H37), a subsample from the same cremation were sent to RICH and the Czech Radiocarbon Laboratory (CRL, Prague, Czech Republic) for replicated measurements. At CRL, cremated samples were pretreated following the standard protocol outlined in Brock et al. (2010) and Snoeck et al. (2016a). Samples were then measured on a MILEA (Multi-Isotope Low-Energy AMS).

All raw dates were calibrated using OxCal v4.4 (Bronk Ramsey 2001, 2021) with the calibration curve IntCal20 (Reimer et al. 2020).

## Results

### Overall results of strontium isotope analysis

**Fig. 3:**
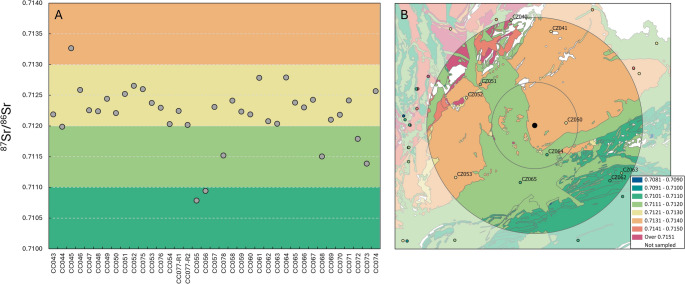
(**A**) ^87^Sr/^86^Sr for all human samples. CC075, CC076, CC077-R1, CC077-R2, CC078 refer to measurements made in teeth, the rest all come from fully calcined long bones. The full list of sample description is provided in S2. (**B)** Location of Přáslavice shown with 20 km and 50 km radii, along with ^87^Sr/^86^Sr plant sampling sites. Background colours relate to the median ^87^Sr/^86^Sr per simplified geological unit, shown further in Fig. 2, with the associated value ranges shown in the legend

The ^87^Sr/^86^Sr results of the modern plant are provided in S3 and plotted in Fig. 2, showing the expected BASr values (as medians) at different geological units. All modern samples measured between 0.7083 and 0.7239, where the “local” (defined as a 20 km radius circle around the site, see Fig. 3B) baseline values are between 0.7106 and 0.7135.

All isotope and infrared measurements and sample information of humans are presented in S2 and summarised in Table 1, where all the human samples have ^87^Sr/^86^Sr values between 0.7108 and 0.7133 and [Sr] range between 75.3 and 165.4. Figure 3 plots all archaeological human samples with modern plant samples. In Figs. 2B and 3B, both the ^87^Sr/^86^Sr median per age lithology category and the ^87^Sr/^86^Sr median per sampling site are depicted. These median values may differ from each other, with sampling site ^87^Sr/^86^Sr depicting the median of grass, shrub, and tree samples, while the age lithology category ^87^Sr/^86^Sr represents all Sr measurements from all sampling sites within that category.

A Shapiro-Wilk test of normality suggests ^87^Sr/^86^Sr data is not normally distributed (*p* = 0.005). Thus, non-parametric tests are used in the following analyses. To ensure comparability of data, only measurements from long bones are considered in the following assessments, unless stated otherwise.

### Date and periodisation of the site

**Fig. 4:**
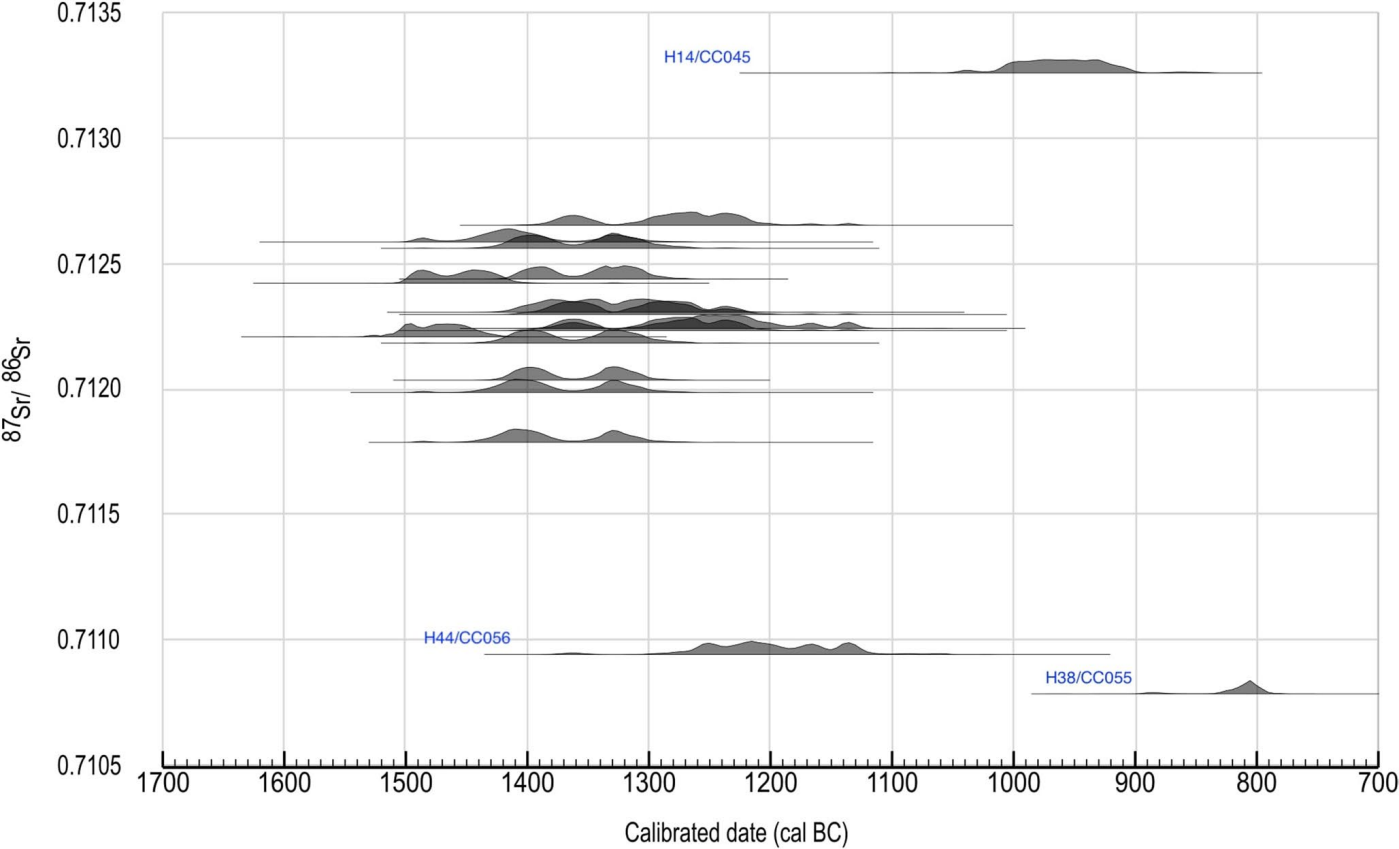
Probability distributions of C14 age (95.4% confidence) of the dated samples plotted against their strontium isotope measurements. Figure created using OxCal v4.4 (Bronk Ramsey 2021)

The results of both uncalibrated and calibrated radiocarbon dates are provided in Table 2. Figure 4 plots the probability distributions of ^14^C age against ^87^Sr/^86^Sr values of the dated samples. All samples provided expected age for Bronze Age burials (1503–786 cal. BCE). Most of the dates fall before 1100 cal. BCE (i.e., Late Bronze Age), with only two burials belonging to the Final Bronze Age: H14 (1047–899 cal. BCE) and H38 (896–786 cal. BCE). Interestingly these two samples also have the highest (0.7133) and the lowest (0.7108) ^87^Sr/^86^Sr, respectively (Fig. 4).

**Table 2:** Radiocarbon dates of all samples from the Přáslavice cemetery, including both original and previously published dates

VUB Lab No.	Grave No.	Fill/layer	Sample	Lab. No.	Date (BP)	Standard deviation	Calibrated (95.4%, BC, AD)	References/comments
	H1	800	charcoal (Acer sp. - maple)	VERA 587	2955	± 45	(-) 1369 − 1015	Šabatová-Vitula 2002
CC044	H7	806	calcined bone, femur	RICH-33,336	3114	± 29	(-) 1446 − 1288	
CC045	H14 I	813	calcined bone, femur	RICH-33,337	2810	± 26	(-) 1047 − 899	
	H14	813	charcoal (Fagus - beech)	VERA 588	2990	± 40	(-) 1386 − 1056	Šabatová-Vitula 2002
CC046	H15	814	calcined bone, femur	RICH-33,338	3132	± 32	(-) 1497 − 1298	
CC048	H25	824	calcined bone, femur	RICH-33,339	3003	± 29	(-) 1381 − 1126	
CC049	H31 I	830	calcined bone, tibia	RICH-33,340				failed
	H31 IIa	830	calcined bone, humerus	CRL 23_1505	3066	± 19	(-) 1406 − 1266	the same bone sample
	H31 IIb	830	calcined bone, humerus	RICH-34,458	3125	± 27	(-) 1493 − 1299	
CC050	H33	832	calcined bone, femur	RICH-33,341	3214	± 26	(-) 1518 − 1427	
CC052	H35	834	calcined bone, femur	RICH-33,342	3023	± 26	(-) 1391 − 1133	
CC054	H37 I	836	calcined bone, femur	RICH-33,343				failed
	H37 IIa	836	calcined bone, humerus	CRL 23_1506	3107	± 23	(-) 1434 − 1294	the same bone sample
	H37 IIb	836	calcined bone, humerus	RICH-34,459	3087	± 27	(-) 1421 − 1272	
CC055	H38	837	calcined bone, femur	RICH-33,344	2648	± 26	(-) 896 − 786	
CC056	H44	843	calcined bone, femur	RICH-33,345	2979	± 27	(-) 1371 − 1113	
CC057	H46	845	calcined bone, femur	RICH-33,346	3065	± 29	(-) 1414 − 1232	
CC059	H51	850	calcined bone, femur	RICH-34,086	3031	± 27	(-) 1397 − 1206	
CC066	H78	877	calcined bone, femur	RICH-34,087	3041	± 27	(-) 1398 − 1218	
CC067	H81	880	calcined bone, femur	RICH-34,088	3180	± 27	(-) 1503 − 1414	
CC070	H91	890	calcined bone, femur	RICH-34,089	3099	± 27	(-) 1430 − 1283	
CC072	H98	897	calcined bone, femur	RICH-33,347	3116	± 27	(-) 1446 − 1292	
CC074	H101	1800	calcined bone, femur	RICH-34,090	3097	± 27	(-) 1428 − 1283	

### Intra-population comparison

No statistically significant correlation can be observed between the ^87^Sr/^86^Sr values of individuals of different sex, age categories, burial practices (i.e. urned, unurned, and mixed), and quality/quantity of grave goods.

### Intra-individual variability

Samples from multiple elements (i.e. bone and dental tissues) were obtained and measured from four of the single burials. Minimal intra-individual variability can be detected and that all individuals have ^87^Sr/^86^Sr values well within the local BASr range (0.7106 and 0.7135; S6).

### Infrared, carbon and oxygen isotope analyses

**Fig. 5:**
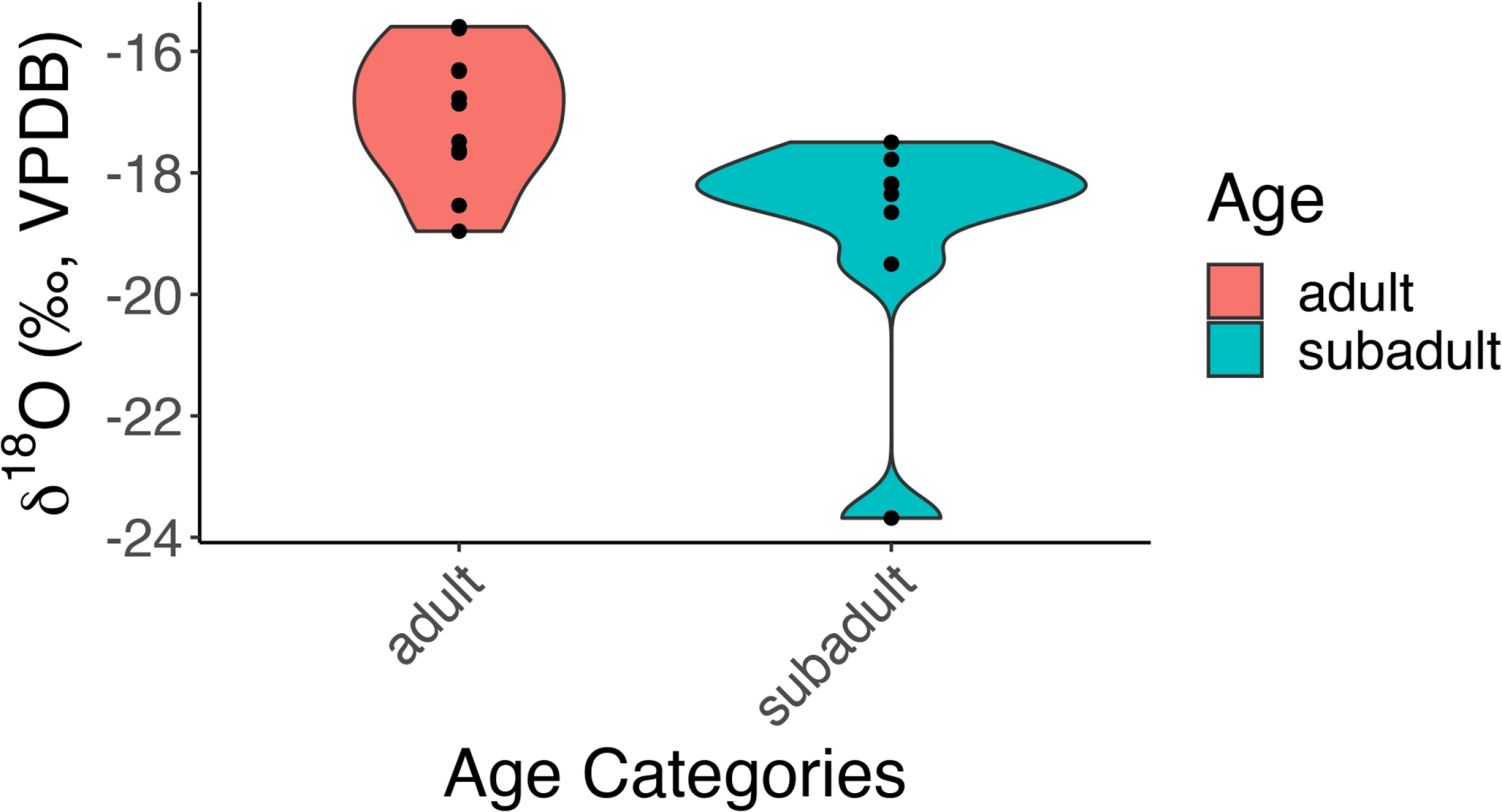
Comparison of oxygen isotope ratios between adults and subadults

The carbon and oxygen isotope ratios as well as the infrared indices (IRSF, C/C, CN/P, OH/P and BPI) of the Přáslavice group show a rather homogenous picture. The only statistically significant difference is observed in the δ^18^O values of the adults compared to the subadults, where the subadults have lower δ^18^O values (Mann Whitney U test; *p* = 0.012; Fig. 5). The difference is still significant after removing the outlier H36 (*p* = 0.024).

## Discussions

### Strontium isotope analysis

Přáslavice is situated at the boundary of two geological units (Miocene and Pliocene sand, gravels, and clays to the west; Carboniferous shales to the east). Local BASr differs between these regions, with ^87^Sr/^86^Sr between 0.7111 and 0.7120 to the east and 0.7131 to 0.7140 to the west (Fig. 3). All analysed burials in this study have ^87^Sr/^86^Sr values (0.7108–0.7133) fall within the local BASr range (0.7106–0.7135), suggesting that all individuals could be local, with little to no intra-site pattern.

While a slight difference can be observed in the distribution of ^87^Sr/^86^Sr between the adults and subadults, the difference is not statistically significant and is likely skewed by the ^87^Sr/^86^Sr values from the two individuals dated to the Final Bronze Age (H14 – a subadult and H38 – an adult). It is important to note that, given the generally small sample sizes of these intra-population groups (*n* < 15), the null hypotheses should be regarded with caution. Additional data from similar sites may provide further insights into the internal social dynamics of Urnfield societies.

All analysed individuals can be considered local, three burials (H14, H38, and H44) have ^87^Sr/^86^Sr values outside the 2 standard deviations of the group, and thus can be considered as outliers. Note that two out of the three outliers (H14 and H38) are the only two burials dated to the Final Bronze Age (1100–800 BCE). Other than simply suggesting these as “immigrants”, one other possibility is a shift in landscape use towards the Final Bronze Age, where the population has begun to exploit resources from a different area. Archaeological evidence from excavations have revealed that the residential areas at Přáslavice for the Final Bronze Age phase have shifted (Šabatová and Vitula 2002; Šabatová 2007b; Vránová 2013). While the shift was only within a 1–2 km radius, this shift has nevertheless implied changed land use towards the Final Bronze Age, which could be linked to other larger changes, such as modes of resources exploitation, that is not yet discovered. A third possibility is that these individuals did not live locally, but were buried at Přáslavice. Earlier studies have suggested that some larger Urnfield cemeteries were “communal” cemeteries that served multiple nearby settlements, interring individuals who, even though did not reside at a particular site during their lifetime, were buried there for cultural reasons (Smrž 1998; Jiráň 2002; Jiráň et al. 2013a). However, none of these hypotheses can be confirmed with our current sample size.

Archaeological evidence indicates connections between Přáslavice with regions 50 km to the north and beyond, including imported raw lithic materials from the Hrubý Jeseník (High Ash Mountains) (Šabatová and Vitula 2002). However, this connection is not supported by Sr values of the humans. Furthermore, the homogenous ^87^Sr/^86^Sr values of individuals from the earlier phase of this cemetery (before 1100 cal. BCE; Fig. 4) support the hypothesis that Urnfield Culture at this site was likely a local development, rather than the result of an incoming flux of newcomers. The buried population at this site exhibits very homogeneous ^87^Sr/^86^Sr values (MAD = 0.0003; Table 1), indicating both low residential mobility and minimal intra-individual variability. Interestingly, the ceramics and bronze artefacts from Přáslavice share many similarities with those from sites in Polish Silesia, such as Kietrz and Zbrojewsko (Gedl 1979; 1999). The lack of agreement between the ^87^Sr/^86^Sr values of the humans and artefact styles suggests that the mode of cultural contacts and exchanges between Přáslavice and the northern Urnfield regions was complex, possibly indirect and independent of significant human mobility. Further discussion on this topic would require additional lines of archaeological evidence for a complete understanding.

The findings of this study generally align with findings from other Urnfield cemeteries in Central and Southern Europe, where strontium isotope measurements of cremated remain indicate that the majority of the population were local. For example, strontium isotope analyses on the cemeteries of Ljubjana Dvorišče (Slovenia), San Valentino (Italy), and Vollmarshausen (Germany) suggest that most individuals were local (Jernejčič and Price 2020; Taylor et al. 2020; Capuzzo et al. 2024). However, it is noteworthy to point out that a recent study on Inzersdorf (Austria), about 180 km south of Přáslavice, revealed some intra-population differences (Fritzl et al. 2024). Fritzl et al. reported that at Inzersdorf, females appeared to exhibit a wider range of strontium isotope values than males, and that comparing to the adults, the subadults tend to display slightly elevated values that align closer with the local baseline. Chronological variation is also evident at Inzersdorf, where individuals dated to the earlier phase show a broader range of values, while the later phase is characterized by more local and homogenised isotope signatures. Inzersdorf, being a much bigger burial site (up to 273 graves), was likely more socially complex. This polarising observation suggests that in the future, we should focus on comparing sites of similar sizes to help control variables in our analysis.

### Carbon and oxygen isotope analysis and FTIR

**Fig. 6:**
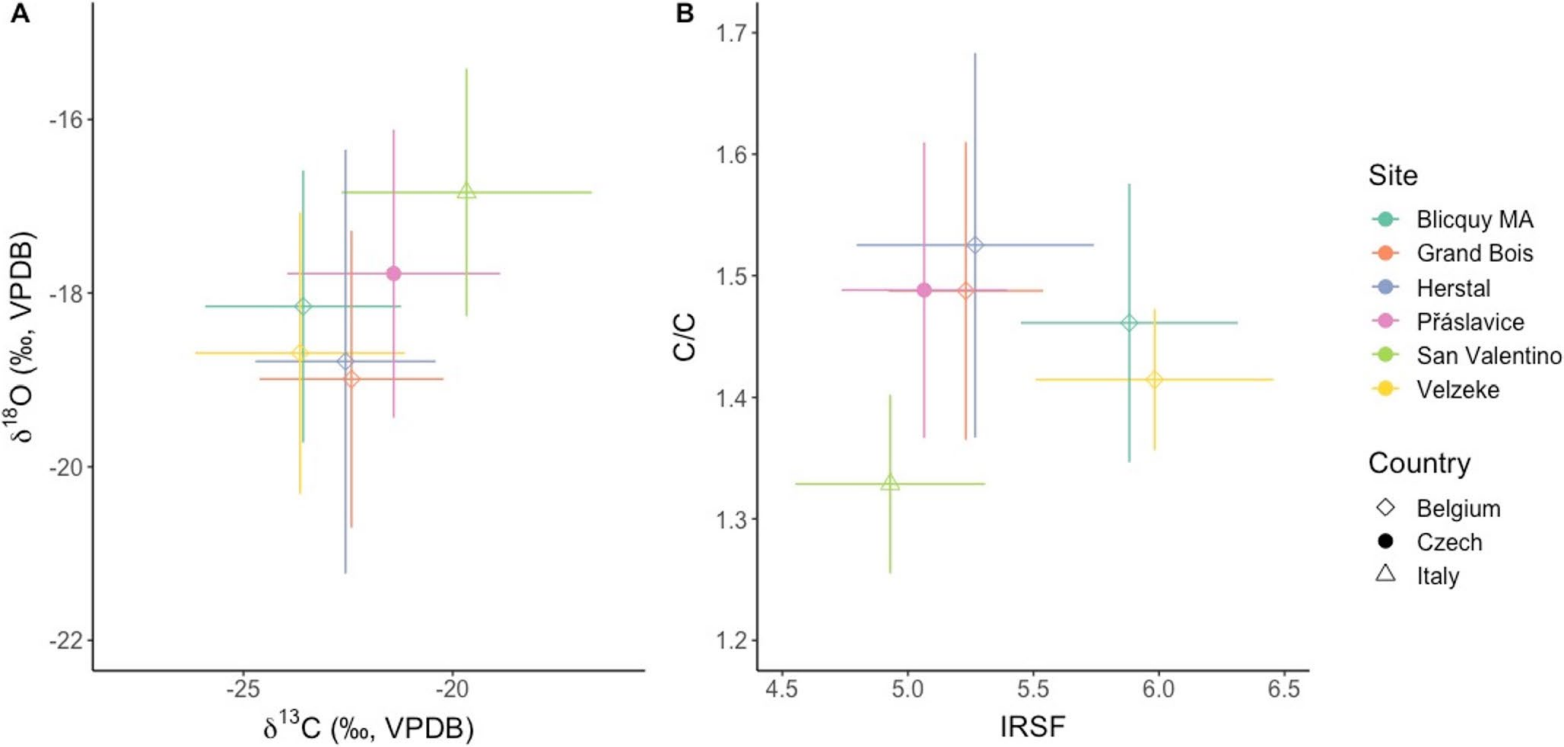
Biplots showing the means and 1SD ranges of the results from: **A**). carbon and oxygen isotope analysis; and **B**). FTIR analysis

The carbon and oxygen isotope ratios as well as the infrared indices confirm the selected samples are fully calcined and burned at temperatures above 650–700 °C. When compared to results obtained from Bronze Age and Iron Age cremated human remains in Belgium (Stamataki et al. 2021) and Italy (Capuzzo et al. 2024), the variability is similar, suggesting a similar level of specialisation (or lack thereof) between the different sites (Fig. 6). The mean values of the carbon and oxygen isotope ratios of the samples from Přáslavice fall between the values seen in Belgium and Italy. While this comparison may not seem immediately significant due to the large geographic span of the sites, the increasing number of studies collecting δ^13^C, δ^18^O, and FTIR data from cremated remains may elucidate clearer trends in the future. One interesting observation is the difference in δ^18^O values observed between the adults and subadults (Fig. 5). A similar observation was also seen in Herstal, a Late Bronze Age/Early Iron Age site in Belgium (Stamataki et al. 2021). As mentioned earlier, δ^18^O in calcined bone can no longer be used to infer lifehistory, but rather, reflects the conditions of cremation (Snoeck et al. 2016b; Salesse 2021; Stamataki et al. 2021, 2025a, 2025b). Therefore, similar to Hertstal, the observed difference in δ^18^O values between adults and subadults at Přáslavice also likely reveal the different conditions adults and subadults were subjected to during the cremation process (e.g. adaptation in pyre size to body dimensions, variations in pyre structure, and other related factors).

The overall results from Přáslavice suggested that the population likely had a local origin and limited mobility during life, and practiced similar cremation procedures to Urnfield sites in other parts of Europe.

## Conclusions

Traditionally, very limited biological information can be derived from cremated remains. This study underscores the potential of a multi-proxy approach in studying cremated remains from the past. In this study, the ^87^Sr/^86^Sr values of the Přáslavice population were found to be highly homogeneous, indicating that the population exploited local food sources. Thus, despite finding artefacts with strong connections with the northern regions, ^87^Sr/^86^Sr data suggests that the Early Urnfield population at Přáslavice was predominantly local. These findings generally align with those from other Urnfield cemeteries in Central Europe, where the majority of the population is presumed to be local. The results of the FTIR and oxygen and carbon isotope analyses also indicate that the funerary rite at Přáslavice did not differ significantly from those observed at other Central European sites.

The results of radiocarbon dating indicate that the majority of the burials are dated to the Late Bronze Age (1300–1100 BCE). This date is consistent with the typological range of cultural materials from the residential area. Two individuals (H14 and H38) dated to the Final Bronze Age (c. 1100–800 BCE) further confirm that the Přáslavice cemetery was used continuously over a long period of time, in spite of shifting settlement areas. In addition, these two individuals also have outlying ^87^Sr/^86^Sr values, suggesting that, towards the Final Bronze Age, there were either changes in population composition, a shift in land use, or that the cemetery was used by a wider group of people. While it is not possible to determine the exact reason behind this, our results raise interesting research questions for future studies to explore. Nevertheless, this study represents a small regional study with limited sample size. Therefore, it is challenging to draw more concrete conclusions from our current findings. However, as additional data from the region accumulates, we hope to interpret these results with greater clarity in the future. Additionally, this study contributes important bioavailable strontium (BASr) baseline data for the region, providing a valuable foundation for future palaeomobility studies to build upon.

## Supplementary Information

Below is the link to the electronic supplementary material.

Supplementary Material 1

Supplementary Material 2

Supplementary Material 3

Supplementary Material 4

Supplementary Material 5

Supplementary Material 6

## Data Availability

All original data reported in this study has been deposited in a public data repository IsoArcH and is openly available at https://doi.org/10.48530/isoarch.2024.004 under a CC- BY 4.0 license following the FAIR and CARE principles (Salesse 2018; Plomp 2022).
