# Supplementary figures and images for: Determining the roots of Urnfield Culture at Přáslavice, Czech Republic
Source: Archaeol Anthropol Sci. 2026 Apr 7;18(5):91. doi: 10.1007/s12520-026-02436-2 (PMC13056762; doi:10.1007/s12520-026-02436-2)

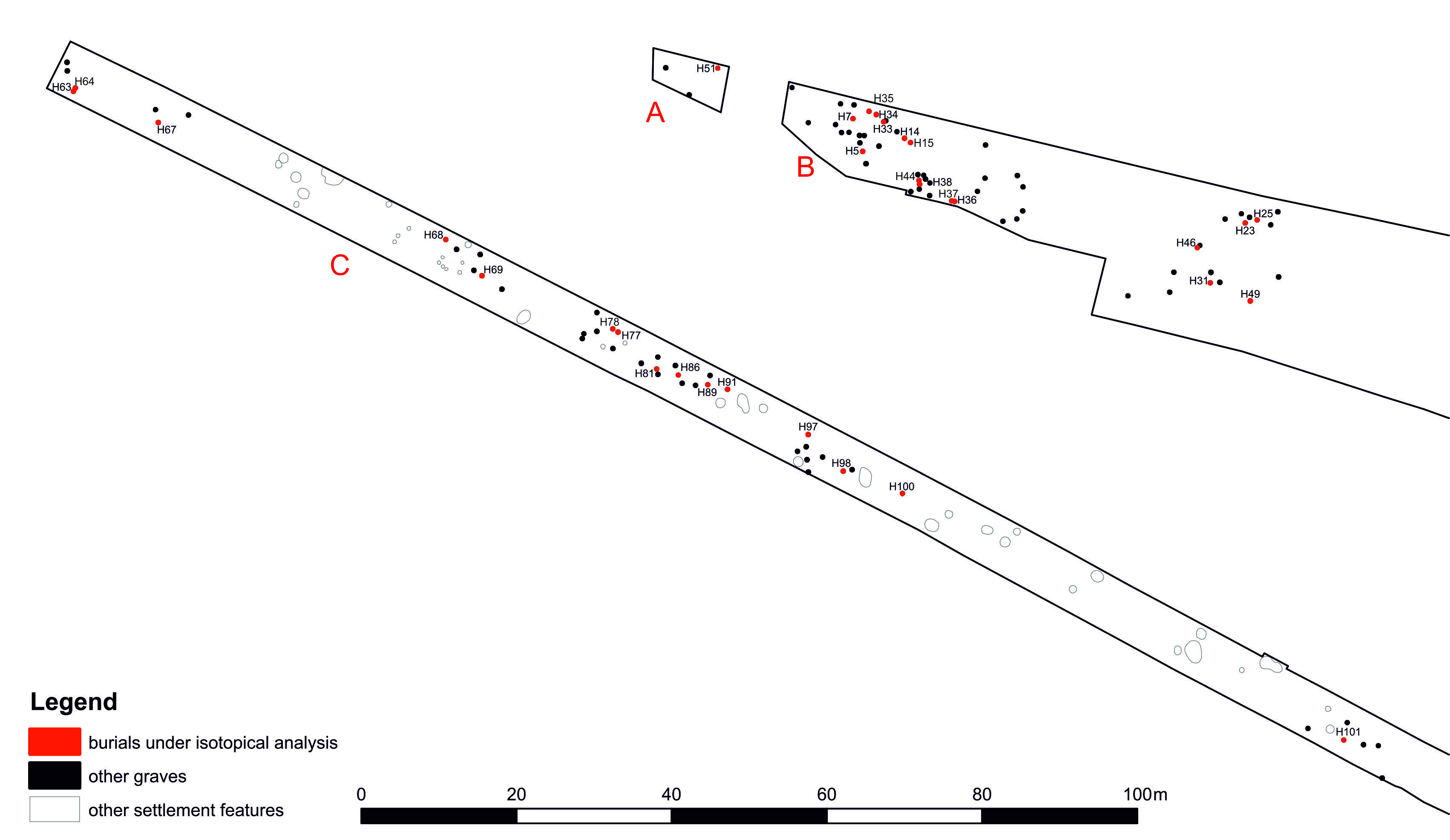

Supplement: Supplementary file 1 — Supplementary Material 1 [file 12520_2026_2436_MOESM1_ESM.jpg]
