# Supplementary material for: Determining the roots of Urnfield Culture at Přáslavice, Czech Republic
Source: Archaeol Anthropol Sci. 2026 Apr 7;18(5):91. doi: 10.1007/s12520-026-02436-2 (PMC13056762; doi:10.1007/s12520-026-02436-2)
Supplement: Supplementary file 4 — Supplementary Material 4 [file 12520_2026_2436_MOESM4_ESM.docx]

# S4 – Detailed descriptions of sample pretreatment and strontium extraction protocols

All samples are treated and processed following well-established protocols described in published reports.

For human samples, a small chunk (100–250 mg) of fully calcined bone was selected from each individual. Each sample was first mechanically cleaned to remove surface contaminants. The samples were then ultrasonicated in milliQ water for 10 mins, and repeated for at least two more rinses, or until the water appeared clear. The samples were then sonicated in 1M CH_3_COOH for 10 mins, followed by three rinses in milliQ water (10 mins in ultrasonicator per rinse). The cleaned samples were then dried in an oven at 50˚C for overnight, and powdered with an agate mortar and pestle. For enamel pieces, about 20–30 mg of materials was used. Enamel was pretreated with 0.1M CH_3_COOH for 30 mins then rinse three times with miliQ water. To prepare the samples for Sr extraction, about 15–20 mg of bone or enamel powder were weighed out and treated with 14 M HNO_3_ at 90˚C overnight. The samples were then left to dry on a hotplate.

For plant samples, each sample consisted about 0.28g of plant materials. Prior to Sr extraction, 4.5mL of 14 M HNO_3_ and 0.5 mL HF were added to each sample, and digested with a microwave (Milestone ultraWAVE). After digestion, 0.5 mL H_2_O_2_ was added to each sample, and samples were left to dry on a hotplate for overnight.

All dried samples (both cremated bone/tooth, enamel, and plants) were processed in a class 1000 clean room. The samples were taken up in 2.5 mL of 7 M HNO_3_ (to ensure all samples were fully dissolved, the vials were sonicated for 20 mins). The samples were loaded onto columns filled with precleaned TrisKem SR Spec resin. For Sr extraction, 4 mL of 7 M HNO_3_ was used to rinse out the samples. Finally, the Sr fraction from the samples were eluted with 6mL of 0.05 M HNO_3_. International standards (NIST SRM 1400, 1486, and 1515) and blanks were processed alongside the samples. The eluted samples were then dried on a hotplate overnight.
