# Supplementary material for: Determining the roots of Urnfield Culture at Přáslavice, Czech Republic
Source: Archaeol Anthropol Sci. 2026 Apr 7;18(5):91. doi: 10.1007/s12520-026-02436-2 (PMC13056762; doi:10.1007/s12520-026-02436-2)
Supplement: Supplementary file 5 — Supplementary Material 5 [file 12520_2026_2436_MOESM5_ESM.docx]

Table S5 Simplified lithology categories from the ‘name’ category from the Geological Map of the Czech Republic 1:500,000 (GEOCR500).

| **Simplified category** | **Includes:** |
| --- | --- |
| Carbonate | Limestone, calcareous claystone, marlstones |
| Sedimentary clastic | Clays, silts, sands, gravels, shales, claystone, sandstone, conglomerate, greywackes |
| Extrusive igneous | Basalt, rhyolite, andesite |
| Intrusive igneous | Granite, diorite, tonalite, gabbro, syenite, teschenite, picrite, peridotite |
| Metamorphic | Gneiss, granulite, phyllite, mica schist, paragneiss |
| Metamorphic igneous | Greenstones, metagranodiorites, orthogneisses |
| Metamorphic extrusive igneous | Amphibolite |
| Metamorphic intrusive igneous | Metagranites |
| Metamorphic sedimentary | Marble, quartzite |
