# Supplementary material for: Determining the roots of Urnfield Culture at Přáslavice, Czech Republic
Source: Archaeol Anthropol Sci. 2026 Apr 7;18(5):91. doi: 10.1007/s12520-026-02436-2 (PMC13056762; doi:10.1007/s12520-026-02436-2)
Supplement: Supplementary file 6 — Supplementary Material 6 [file 12520_2026_2436_MOESM6_ESM.docx]

Figure S6 Intra-individual ^87^Sr/^86^Sr data. Dotted lines correspond to the local bioavailable strontium baseline range.
